# Nursing-Related Barriers to Children's Pain Management at Selected Hospitals in Ghana: A Descriptive Qualitative Study

**DOI:** 10.1155/2020/7125060

**Published:** 2020-01-20

**Authors:** Abigail Kusi Amponsah, Evans Frimpong Kyei, John Bright Agyemang, Hanson Boakye, Joana Kyei-Dompim, Collins Kwadwo Ahoto, Evans Oduro

**Affiliations:** ^1^Department of Nursing, Faculty of Allied Health Sciences, Kwame Nkrumah University of Science and Technology, Kumasi, Ghana; ^2^Department of Nursing Sciences, Faculty of Medicine, University of Turku, Turku, Finland; ^3^Asonomaso Government Hospital, Kwabre East–Ashanti, Ghana; ^4^Department of Nursing and Midwifery, University of Limerick, Limerick, Ireland; ^5^Institute of Public Health and Clinical Nutrition, University of Eastern Finland, Joensuu, Finland

## Abstract

Staff shortages, deficient knowledge, inappropriate attitudes, demanding workloads, analgesic shortages, and low prioritization of pain management have been identified in earlier studies as the nursing-related barriers to optimal children's pain management. These studies have mainly been undertaken in developed countries, which have different healthcare dynamics than those in developing countries. The current study, therefore, sought to identify and understand the nursing-related barriers to children's pain management in the Ghanaian context. A descriptive qualitative study was conducted among 28 purposively sampled nurses working in the pediatric units of five hospitals in the Ashanti region of Ghana. Over the course of three months, participants were interviewed on the barriers which prevented them from optimally managing children's pain in practice. Recorded interviews were transcribed verbatim and deductively analysed based on a conceptual interest in pain assessment and management-related barriers. NVivo 12 plus software guided data management and analyses. The mean age of participating nurses was 30 years, with majority being females (*n* = 24). Participants had worked in the nursing profession for an average of five years and in the pediatric care settings for an average of two years. The nursing-related barriers identified in the present study included communication difficulties in assessing and evaluating pain management interventions with children who have nonfunctional speech, insufficient training, misconceptions on the experience of pain in children, lack of assessment tools, and insufficient number of nurses to manage the workload and nurses' inability to prescribe analgesics. The present study revealed some barriers which prevented Ghanaian nurses from optimally managing children's pain. Nurses should be educated, empowered, and supported with the requisite material resources to effectively manage children's pain and improve outcomes for families, healthcare systems, and the nation. Future studies should explore the facilitators and barriers from other stakeholders involved in pediatric pain management.

## 1. Introduction

Pain is a major source of distress for hospitalized children, family caregivers, and healthcare providers alike [1]. Pain in children can result from physical damage, disease process, invasive procedures, and other unknown factors. The effects of unrelieved or poorly managed children's pain cannot be overemphasized. Apart from the short-term bio-psycho-socio-developmental effects it has on the child in pain [2, 3], it can also lead to altered pain sensitivity and chronic pain which cost billions of dollars to treat and become a source of burden for families, healthcare systems, and nations [4]. All these undesirable consequences underscore the need for prudent management of children's pain.

Nurses remain key players in medical care as they form most of the healthcare providers and spend the most time with hospitalized children and their families. Thus, they are well positioned to directly and indirectly affect all aspects of children's pain management. Nevertheless, there are several impediments that prevent nurses from optimally managing children's pain. A cross-sectional survey of nurses in the United States revealed insufficient medication orders by physicians, inappropriate timing of premedications, low prioritization of pain management [5], and delays in analgesic availability [6]. A qualitative study in the United Kingdom found shortage of staff, knowledge deficits, demanding workload, and pain medication shortages as the nursing-related barriers to optimal pain care in children [7]. Aziznejadroshan and colleagues report of improper organizational structure, disruption of pain management activities, insufficient nursing competency, undesirable characteristics of children and their parents, and inefficient companions as the nursing-related barriers to pediatric pain management in a qualitative study conducted in Iran [8].

Earlier studies have been carried out in both developed and developing countries, which have different healthcare dynamics than those in the Ghanaian context. Exploring and validating the barriers in resource-limited healthcare environments would be useful in directing strategies that could enhance children's pain management. The current study, therefore, sought to unearth the nursing-related barriers to children's pain management in the Ghanaian context.

## 2. Methods

### 2.1. Study Design and Setting

A descriptive qualitative study was carried out in five hospitals in Ghana over a three-month period. This study formed part of a larger project that examined the educational needs of nursing students and nurses on pediatric pain management. The included hospitals were chosen as they represented diverse healthcare facility categorizations (private, government, quasigovernment, and missionary) in urban, periurban, and rural areas. A quasigovernmental hospital is a hospital that has the characteristics of both government and private institutions [9]. It can thus refer to a government hospital which is managed privately by an organisation or a privately owned hospital which is managed by a government institution. Two of the hospitals were specialist children's hospital, while the remaining three had a children's department within the hospitals' structure (Table 1).

The in-patient bed capacities within the pediatric care settings of the included hospitals extended from eight to 50 with a mean of 23 beds. The range of nursing staff in these hospitals was five to 15 with a mean of 11 nurses. These nurses were responsible for managing the in-patient pediatric care settings on a three-tier shift system (morning, afternoon, and night). On average, 10 children were admitted to these hospitals on a weekly basis. Nursing activities in these settings consist of health assessment, planning, implementation, and evaluation of basic and advanced healthcare interventions. Pain assessment and management of hospitalized children are expected of the nurses working in these hospitals.

### 2.2. Participants

Nurses working in the children's unit of the selected hospitals for a minimum of two months were eligible for participation in the current study. Twenty-eight (28) nurses with varied working years in the children's department were purposively sampled over a three-month period (October–December 2018). Theoretical sampling saturation was achieved within and across the hospital groups.

Participating nurses comprised of 24 females and four males; their ages ranged from 24 to 38 years with a mean age of 30 years. Upon graduating, participants had worked in the nursing profession for an average of five years (range of six months to 14 years). However, they had worked as nurses in the pediatric settings for an average of two years (range of two months to 10 years). Participants had obtained certificate (*n* = 5), diploma (*n* = 16), bachelor's degree (*n* = 5), and master's degree (*n* = 2) as their highest educational nursing qualification. The nurse participants worked in a private specialist children's hospital (*n* = 5), a government specialist children's hospital (*n* = 5), a government general hospital with a pediatric department (*n* = 7), a quasigovernmental general hospital with a pediatric department (*n* = 6), and a missionary general hospital with a pediatric department (*n* = 5).

### 2.3. Data Collection Procedures

Upon administrative and ethics approval from the relevant authorities, hospital administrators or nurse managers within the hospitals led the research team and introduced them to the target group of nurses who were in the pediatric care settings. The participants were then informed about the aim and procedures involved in the study before consenting to participate. A scheduled date, time, and venue were agreed by both the researchers and eligible consenting participants for the interviews to be held.

Individual and group interviews were conducted on scheduled dates to improve procedural integrity and rescheduled in some instances due to tight working plans of some participating nurses. The interviews were recorded with participants' permission using the English language and lasted from 10 to 30 minutes per each session. With the aid of a semistructured guide, four (AKA, JKD, CKA, and EO) of the researchers who had been trained in the conduct of qualitative research facilitated the interview sessions at the respective hospitals. A minimum of two researchers were present during each interview to allow for notes taking and the smooth running of the sessions. At the end of each interview session, participants were briefed about the main points discussed during the session for clarifications and corrections as deemed necessary. All interviews were completed in one hospital before moving to the next. Participants were given the opportunity to recount the obstacles which prevented them from optimally assessing and managing children's pain in practice (refer to Appendix A for the interview guide used).

### 2.4. Analysis

Each recorded interview was transcribed verbatim and checked for correctness by the participants and at least two members of the research team. Subsequently, the transcript was analysed before moving on to the next interview session. All the researchers involved in the current study independently read the transcripts several times and coded them for their basic units of meaning. The team met several times to deliberate on the generated codes and resolved discrepancies through consensus building. The codes were then combined to form larger meaningful units using deductive analytic techniques until the agreed themes were actively generated. NVivo 12 plus software guided the management and analyses of the qualitative data.

### 2.5. Ethical Considerations

Administrative approvals for the study were granted by the management of the respective hospitals. Ethical approval (reference number: CHRPE/AP/574/18) was also given by the Committee on Human Research, Publications, and Ethics (CHRPE), School of Medical Sciences (SMS), KNUST, Ghana. Ethical principles such as autonomy, privacy, confidentiality, justice, and voluntary participation were adhered to during the entire research process. Additionally, participants were informed that they could withdraw at any time from the study without any negative consequences. Participants' data were accessible only to the researchers involved in the study; thus, no identifiable information was used during the reports of the study findings.

### 2.6. Trustworthiness

Lincoln and Guba's principles of credibility, confirmability, dependability, and transferability were adhered to as measures of ensuring trustworthiness in the present study [10]. Member checking of transcripts by the participants, checking the transcripts for correctness by the researchers, and peer checking of generated themes by an experienced qualitative researcher served as a means of ensuring credibility and confirmability of the findings. Detailed field reports, description of the study settings, and processes involved in the entire study also served as methods of enhancing dependability and transferability of the study findings.

## 3. Findings

### 3.1. Nursing-Related Barriers to Pediatric Pain Management

**Figure 1:**
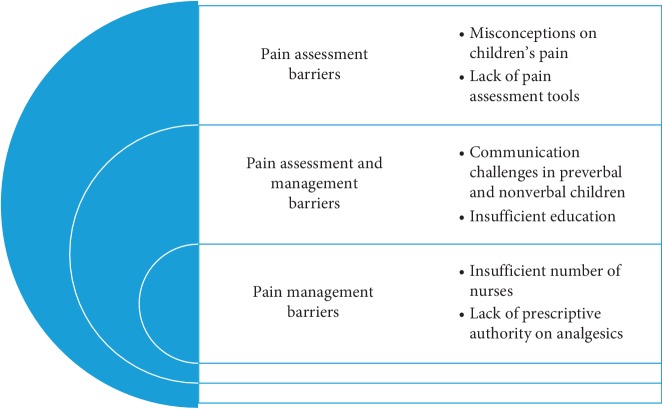
Visual representation of identified pain assessment and management barriers.

Participants recounted some impediments which they described as barriers which prevented them from assessing and managing children's pain effectively. Among them were communication challenges, insufficient training, misconceptions on children's pain, lack of pain assessment tools, inadequate nursing staff, and lack of prescriptive authority on analgesics. These barriers have been grouped into pain assessment and management, respectively (refer to Figure 1).

### 3.2. Pain Assessment-Related Barriers

Participants narrated some obstacles which they believed prevented them from optimally assessing children's pain. They recounted communication challenges in pain assessment due to some children who were not able to communicate verbally or had insufficient vocabulary to describe their pain. They therefore relied on other behavioural indicators (such as facial expressions, body movements, crying, and consolability) or physiological parameters (increases in temperature, pulse, respiration, and blood pressure) which were not exclusive to pain and could be due to several other causes. They believed this affected their pain management behaviours due to difficulties they encounter in assessing the pain of these children.

  For the assessment, it's kind of difficult… especially for those who can't verbalize that they are in pain because we've not received any training on how to assess pain, it's kind of difficult but once you don't know that the child is in pain, how do you manage? hmmmm (Participant 3, Hospital A)
  And sometimes when they are crying, you wouldn't know if it is because of pain or because of feed or because of anything… sometimes too they exaggerate, they just exaggerate that they are feeling pain (Participant 5, Hospital B)

Participants also attributed their pain assessment inadequacies to their insufficient training in the area. They harbored some misconceptions which affected their assessment of children's pain and attributed their suboptimal pain assessment behaviours to the absence of pain assessment tools in the children's unit.

  You know our part of the world… we even don't believe kids, especially babies, that they do experience pain and that crying is their language so, I mean it's normal for a baby to cry. (Participant 4, Hospital C)
  Sometimes when the children say, I feel pain here, I feel pain here, we are just assumed, just assumed, especially during the procedure… just assumed they don't want to have the procedure… also, we don't have any assessment tool in this unit (Participant 1, Hospital E)

### 3.3. Pain Management-Related Barriers

The nurse participants recounted a number of factors which they deemed as barriers that prevented them from effectively managing children's pain. Participants believed the inadequate number of nursing staff to manage the patient load and the time-demanding nature of some nonpharmacological pain management interventions prevented them from optimally managing children's pain. They also attributed their pain management deficiencies to insufficient training, communication challenges in evaluating pain management interventions among children with nonfunctional speech, and the fact that they do not have the authority to prescribe analgesics.

  The patient ratio to nurse ratio is not all that good… You wouldn't have time. You have a lot of work to do and here we are dealing with kids, we work with time so by the time a child is complaining of something, maybe you are doing something, you attend to another child so you can't get enough time to do the diversional therapy for the child… in school we were just taught the theory part… but if it was demonstrated to us to have practised it in school, I think when we come to the field we will be able to… because we were just taught the theory part, there was no practice, there was no demonstration, so it will be very difficult to do, it takes time and we, we've not been in the ward for so long so we are now trying to learn from our senior colleagues so I pray and hope that maybe as time goes on, we will be able to do that…. You know… it's lack of education on the (pain management) interventions (Participant 2, Hospital C)
  So, it is the evaluation of pain management interventions being a problem because they cannot speak and so the healthcare providers have to evaluate it themselves. So I don't think it is adequately managed…It is very difficult when it comes to evaluating pain management interventions (Participant 3, Hospital E)
  As a nurse, you can do your part… It's not us who prescribe drugs so mostly your own is therapeutic ones like diversion therapy unless you call them (doctors) for them to give you go ahead and give this… do you get me? … You can't get up and give a drug without the doctor's advice. So everything you have to consult (Participant 1, Hospital D)

## 4. Discussion

The study explored nursing-related barriers to children's pain management at five selected hospitals in Ghana. Our findings revealed that several pain assessment and management-related factors serve as impediments to optimal pain management by nurses.

Consistent with previous studies [11, 12], ineffective communication with children who have nonfunctional speech was identified as a nursing-related barrier to pediatric pain assessment and management evaluation in the current study. This situation results from some children's inability to describe the presence, location, and extent of their pain due to their developmental stage and limited range of the subjective pain experience [13]. This often creates pain assessment difficulties as nurses have to rely on unreliable behavioural and physiological indicators as self-report is the gold standard of pain measurement [14]. Ultimately, this imparts on pain management and the evaluation of interventions directed toward pain relief, similar to the findings of earlier studies [15–17]. Augmentative and alternative communication approaches (such as communication boards, drawings, speech-generating devices, and computers) could also be used for children with nonfunctional speech due to possibility of misinterpretation of their behaviours by others [18–20].

Nurses in the current study reported of insufficient training as a major barrier to their optimal assessment and management of children's pain. This finding is similar to those reported by recent studies as a major obstacle for optimal pediatric pain management [21–23]. Some of the reasons for the insufficient pediatric pain education in Ghana can be attributed to the limited information received on pain during training as well as limited postgraduate training opportunities where pain is addressed [24]. It may also be possible that nurses who are involved in continuous educational programs after their first qualification do not share this knowledge with other nurses who might not have the opportunity to enroll in these programs. This finding may be responsible for the suboptimal pediatric pain management in practice and calls for more collaboration of knowledge among nurses [25]. Practicing nurses would benefit from additional regular training on pediatric pain management in order to improve outcomes for vulnerable children and their families.

Misconceptions on the pediatric pain experience were also identified as a barrier to optimal pain assessment in the present study, similar to other studies [14, 26]. These misconceptions by health workers especially nurses usually delay effective pain management. It makes them feel reluctant in addressing their pain concerns and makes children experience undue pain. As advocated for by Von Bayer, it is important for all healthcare workers to accept the existence of pain in children to ensure optimal relief [17].

One of the barriers to children's pain assessment was the absence of assessment tools in practice, which is similar to earlier findings [27, 28]. Dürango et al. aver that, for effective and optimal pain management [29], it is important for healthcare providers to select and employ appropriate pediatric pain assessment tools. It is therefore important for nurses to select and use appropriate assessment tool for pain management to enhance optimal pain relief [30]. Previous studies [21, 31] report of lack of pain assessment tools which were linked to suboptimal pain management of patients. Although any such linkages were beyond the scope of the present study, we believe the absence of pain assessment tools may negatively impact pediatric pain management as pain assessment tools serve as a guide in providing baseline information and serving as a means of evaluating the effectiveness of pain management interventions [32].

Inadequate nursing staff to manage the patient workload was reported by the interviewed nurses as one of the barriers to optimal pediatric pain management. This finding is similar to those reported by earlier research studies [8, 32–35]. This situation often leads to stress and burnout among nurses, thereby disenabling them to effectively discharge their duties including pain management. While efforts to increase the nursing personnel should be strengthened, parents and family caregivers should also be adequately prepared for their role in assessing and managing children's pain so that nurses are not overburdened in solely being responsible for this task. Pain management is a shared responsibility and requires the support of all stakeholders to improve outcomes [36].

Lack of prescriptive authority on analgesics by nurses was also revealed as one of the pediatric pain management barriers in the current study. The nurse participants indicated they did not have the authority to prescribe analgesics which often led to the lack of child-family confidence in their abilities to deal with reported pain complaints. Some studies have shown that some level of analgesic prescriptive authority enhances nurses' confidence and autonomy in patient care [37, 38]. Nurses spend most of the time with hospitalized children and their families; it is therefore imperative to empower them for optimal pain management. Nonpharmacological pain management techniques should also be employed in reducing children's pain and the amount of analgesic medications that may be required in treating pain due to their associated side effects [39]. Examples of such techniques include breastfeeding, oral glucose solution, deep breathing exercises, video games, television, music, distraction, and guided imagery, among others [13, 40].

### 4.1. Strengths and Limitations

One of the strengths in the current study is the use of a qualitative research methodology in obtaining detailed information about the barriers that prevent nurses from optimally managing children's pain in pediatric care settings. Akin to all qualitative studies, the results of this study cannot be generalizable but can be transferable to other similar settings. One of the shortfalls of the present study is that we did not explore the facilitators of optimal pediatric pain management. Future studies should consider both the facilitators and barriers to optimal pediatric pain management from other stakeholders such as physicians, healthcare systems, children, and their family caregivers. Despite the abovementioned shortfalls, the present study provides useful insights and enhances our understanding on the nursing-related barriers to effective pediatric pain management.

## 5. Conclusion

The present study revealed some barriers which prevented Ghanaian nurses from optimally managing children's pain. These barriers pertain to communication challenges in assessing and evaluating pain management interventions among children with nonfunctional speech, insufficient training on pediatric pain assessment and management, misconceptions on children's pain, lack of pain assessment tools, shortage of nurses to handle the workload, and nurses' inability to prescribe analgesics. These barriers provide us with an opportunity to improve pain through targeting of interventions capable of overcoming these impediments in practice. Nurses could be educated, empowered, and supported with the requisite material resources to effectively manage children's pain and improve outcomes for families, healthcare systems, and the nation. Future studies should explore the facilitators and barriers from other stakeholders involved in pediatric pain management.

## Figures and Tables

**Table 1:** Characteristics of the study settings.

Hospital	Category	Area	Number of beds	Nursing staff in the pediatric units
A: specialist children's hospital	Private	Urban	15	12
B: specialist children's hospital	Government	Urban	50	15
C: general hospital with a pediatric department	Quasigovernmental	Periurban	22	10
D: general hospital with a pediatric department	Government	Periurban	20	15
E: general hospital with a pediatric department	Missionary	Rural	8	5

## Data Availability

The qualitative data used to support the findings of this study are available from the corresponding author upon request.

## Appendix

### A. Interview Guide

(1) Unit, Hospital: __________
(2) Age: _____
(3) 3. Sex: _____ (a) Female _____ (b) Male _____ (c) Others
(4) Years of Experience in Nursing: ……………………
(5) Years of Experience in Children's Nursing: ………………………
(6) Educational Preparation in Nursing
  - Certificate
  - Diploma
  - Bachelor of Science
  - Associate Degree
  - Masters
  - Others, specify …………………………………………………. .

### B. Main Questions

1. To what extent do the children who are in your care complain about pain? (Probes: Do you have examples of these?)
2. How do you assess children's pain complaints in this unit?
3. To what extent are you satisfied with the assessment of children's pain in this unit?
4. What do you feel prevents children from receiving adequate pain assessment from nurses? (Probes: Do you have examples of these barriers? Can you explain this a little further?)
5. How do you manage children's pain in this unit?
6. To what extent are you satisfied with the management of children's pain in this unit?
7. What do you feel prevents children from receiving adequate pain management from nurses? (Probes: Do you have examples of these barriers? Can you explain this a little further?)
